# Challenging Health Service Delivery Models to Improve Access to Physical Therapy in Rural, Remote and Northern Communities

**DOI:** 10.3389/fresc.2022.892038

**Published:** 2022-06-14

**Authors:** Liris P. R. Smith

**Affiliations:** YukonU Research Center, Yukon University, Whitehorse, YT, Canada

**Keywords:** physical therapy, Yukon territory, health service equity, rural/remote, community engagement, relationships

## Abstract

Effective rural, remote and northern physical therapy services are an important component of health care. Providing these services with limited financial and human resources can present many challenges. Indigenous communities also have unique needs that must be considered when providing health care. Most current service delivery models are based in Western medicine practices and most often, do not account for the local, political, cultural and spiritual needs of communities. In this perspective article, I discuss the challenges of providing these services in rural Yukon to many small First Nation communities. Relationship building is paramount to effective and meaningful health care programs, and this means a change in current practice approaches. We need to challenge the delivery models and be open to other ways of knowing, beyond the Western biomedical approach that is the foundation of our profession. It is imperative that physical therapists, health care providers and funders seek new and innovative ways to provide services to the rural, remote and northern communities while ensuring a culturally humble approach.

## Introduction

I started writing this article while visiting the remote village of Old Crow, the home of the Vuntut Gwitchin First Nation, above the arctic circle in Yukon, Canada. I reflect on a career providing services in communities, as a physical therapist and health manager/director. Now I visit Old Crow, as a researcher, supporting patient-oriented health research at the only Canadian university north of 60, Yukon University.

**Figure 1:**
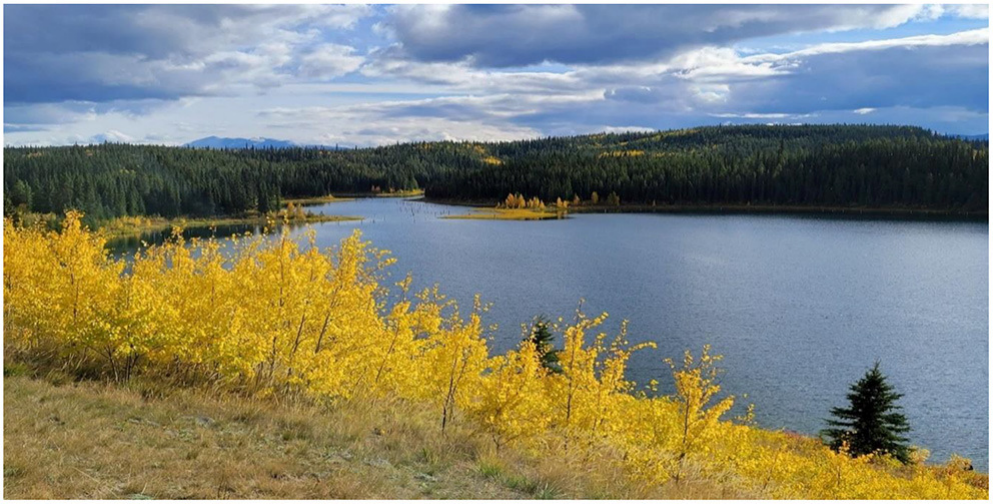
Yukon, Fall 2021.

I have visited almost every community in Yukon (Figure 1) while overseeing rehabilitation service delivery in the remote, rural First Nation communities, using an itinerant model of care. This model is used in northern and remote areas around the globe to increase access to services for people within areas with vast geography and limited resources (1). This model most often involves health care professionals traveling from a larger center to provide services to smaller remote communities, sometimes called Hub and Spoke model (1). Though it may be an accepted model of service delivery, itinerant care has it routes in biomedicine, founded in a Western paradigm that can limit the ability to establish relationships and trust within communities.

New health technologies offer innovative ways to connect with rural and remote areas, but also come with barriers to implementation. One of the primary conditions of success in rural and remote care delivery models is a consistent workforce that can support sustainability, which is not always possible (1). Ultimately, the question is how can we develop true collaborations and work in partnership with communities to improve rehabilitation health outcomes?

First Nation communities in Yukon survived and thrived long before the influx of European settlers and colonization. Canada has a dark and storied colonial past, including the taking of land from Indigenous peoples with subsequent oppression and cultural genocide (2, 3). Many Canadians, like myself, were poorly educated as to the true history of our country, and must seek opportunities to educate ourselves on this important topic. Racism remains evident in this country, at the individual level, but also at the institutional level in the policies and practices of governments. This is also prevalent within the health care field. Even with best intentions, the foundation of health service delivery is primarily Western and Eurocentric. The power imbalances that occur set up barriers to access and may trigger intergenerational trauma for Indigenous people.

## Physical Therapy Services in Rural and Remote Canada

The physical therapy profession has been established in Canada for over 100 years. The profession has developed a strong reputation within the Western health system based on its roots within biomedical philosophies (4, 5). Physical therapy (PT) services have been shown to support quality health care in the management of chronic conditions and disease/injury prevention (6). However, with an historically strong connection of physiotherapy to Western medical philosophies that support dualism (separation of body and mind) and a hierarchical system where the clinician is “expert”, we must look to the broader aspects of health, such as culture, politics, gender and spirituality, in order to meet the needs of those we serve (7).

For Indigenous people, access to PT services in rural and remote communities in Canada varies across the country. There have been movements in Canada to advocate for better access and increased funding to support service provision in remote, rural and Indigenous communities, including cultural training for providers, using elders as advisors for service delivery, accessing translation services, increasing fly-in visits by itinerant therapists and expanding telehealth/telemedicine (6). However, rural, remote and northern communities have small populations that are spread over a large geographical landmass. Bringing a full array of services to people is a challenge in relation to financial and human resources. Additionally, there are challenges in having consistent staff, lack of universal funding models, lack of specific community needs assessments and planning, and lack of culturally appropriate training in providing rehabilitation services (5, 8). Solutions to overcoming barriers to accessing PT services in rural and remote areas in Canada are complex and require engagement with multiple stakeholders (8). The development of relationships in small communities and consistent service is vital to ongoing assessment of needs and provision of service at the individual and community level.

There have been changes in recent years to include cultural safety and humility training in the curriculum of health care professionals in Canada. This is true for rehabilitation professionals as well. Additionally, there is increased training available for practicing physical therapists to learn about the history of Indigenous peoples and the need for change in practice to support cultural safety (9). There is also a trend toward ensuring reflective practice is integrated into the clinical role of rehabilitation professionals and support to identify and challenge systemic racism within the health care system (9–11). These are all positive trends, but must be matched with broader changes to culturally appropriate services with the large health system, rather than relying only on individual changes in practice approaches.

## Emerging Health Care Trends and Service Options

Telehealth and telemedicine service delivery options have been evolving for several decades, though the recent COVID-19 pandemic has accelerated the uptake and use of these technologies (12). For many clinicians, the prospect of alternate ways to provide service and stay connected with clients in rural and remote areas is appealing and exciting. Despite interest and passion of individuals, challenges with other remote/rural health care delivery methods in Canada are mirrored in telehealth and virtual care. The question of equitable access remains, as infrastructure to support connectivity is lacking, and the cost of both hardware and internet access are extremely high in remote areas (13). The maintenance of a therapeutic relationship with clients/patients is also difficult to maintain using telehealth delivery (12). Many remote areas lack resources and training to support uptake of new technologies (13) and other priorities that impact wellness, such as the lack of adequate housing and food security, overshadow the ability to implement digital technologies and digital literacy.

Cultural safety for Indigenous people when accessing virtual care, telehealth and telemedicine is also a concern. A systematic review of Indigenous views of telehealth revealed that many Indigenous people prefer face to face interactions, and strong relationships and trust must first be established for successful implementation of telehealth services (13). In Yukon, the telehealth network began in 2006 with connections between rural community nursing stations and other health services within Whitehorse (14). Though this system has provided opportunities for education, clinical visits and consultation with specialists, the usage has been limited by the location of the units, the need to book through a central coordinator and the fact the equipment was outdated (14). There have been recent upgrades to this technology, as well as adoption of other means of virtual care, such as telephony, video visits using varying platforms and a large investment the 1 Health system that will support an integrated electronic medical record with virtual care capability. The goal of 1Health is to provide a seamless care journey through a single health information system for all Yukoners. These are all very promising approaches and could support the consistent delivery of rehabilitation services in communities. However, the need for relationship-building and establishing trust remains.

## Discussion: Better Service Delivery = Better Relationships

The fact remains that people living in rural, remote and Indigenous communities in Canada need access to rehabilitation services such as physical therapy. Whether these services are delivered in person, virtually, or within a hybrid approach, meaningful relationships and trust are paramount to achieving good health outcomes (8, 10). There is no short cut to this end, as it takes time to build relationships and get to know and understand the needs of individuals and the unique collective culture and norms within each community.

The Western, clinical model of care has not been an effective approach to date, so clinicians, health leaders and funders need to consider new ways of delivering community health services (2, 4). How can we truly integrate the best of Western medicine, with equally efficacious Indigenous ways of knowing and doing? We must find ways to include traditional knowledge by seeking and honoring the wisdom of the knowledge keepers in the community.

Fundamental approaches and elements within our service delivery models need to change to provide culturally relevant and effective services in rural and remote communities. We must move away from imposing a Western view of health and wellness as an individual responsibility. Family and community are integrated into the lives of many Indigenous people and should be a part of their rehabilitation and wellness experiences (10). Rehabilitation professionals have the opportunity to expand their role beyond the biomedical, or even the biopsychosocial, to incorporate cultural and spiritual elements that are inherent in Indigenous knowledge systems (7).

I remember visiting a community early in my PT career and realizing that the most important part of my visit was to advocate, by writing a letter for a client to have basic modifications to their house. The referral was for PT and OT functional assessment, provision of a home exercise program and recommendations of adaptive devices to support independence. However, other issues took priority. This individual, who had a multi-system rheumatoid disease, had to climb onto a sawhorse (a frame that supports wood for sawing) to access their home and did not have access to safe drinking water. The individual and family were not successful in advocating for home adaptions. It was only through an outsider with “expertise” and perceived power that the need was addressed. Though we (OT and myself) felt we made a difference in improving this person's independence, we were not able to fully assess his more specific rehabilitation needs, due to the urgent basic needs of adequate housing and access to water. We need to challenge these systems and advocate for community resources to support the broader needs and health determinants for those living in rural and remote areas of our country. To truly move toward reconciliation, we must decolonize our approaches to providing health services, and balance the power away from the clinician as expert (8).

In a book chapter that critical reflects on physiotherapy practice, we suggested ways for the PT profession to interface with communities, as recommended by our Métis co-authors (10). These recommendations included: being visible and building trusting relationships within the community, being open to cultural practices and engaging in these activities when opportunities arise, learning some words in the local language, sharing your own experience and working collaboratively for open communication, gathering and connecting with the community to share stories and knowledge, and expanding activities and treatment to the land and culture (10). Again, this type of work takes time, along with a willingness from both the health provider and the funder to explore innovative ways of providing services that deviate from the clinical and segmented Western approach. Embedded within a new approach is the fundamental principle of cultural humility, which involves active listening and creating space for new knowledge (10, 11).

I am fortunate to live, work and play on the traditional territories of the Kwanlin Dun First Nation and Ta'an Kwachan council, and to visit the other 12 Yukon First Nation communities that have survived a colonial past, and still hold the knowledge and traditions that support health and wellness of future generations. I believe that the profession of physical therapy has much to offer remote northern communities in Canada, but we must apply this knowledge to the local needs and context and be open to other knowledge systems to create innovative ways rehabilitation services.

## Data Availability Statement

The original contributions presented in the study are included in the article/supplementary material, further inquiries can be directed to the corresponding author.

## Author Contributions

The author confirms being the sole contributor of this work and has approved it for publication.

## Funding

This article was funded through a Scholarly Activity Grant (SAG) through Yukon University, Yukon.

## Conflict of Interest

The author declares that the research was conducted in the absence of any commercial or financial relationships that could be construed as a potential conflict of interest.

## Publisher's Note

All claims expressed in this article are solely those of the authors and do not necessarily represent those of their affiliated organizations, or those of the publisher, the editors and the reviewers. Any product that may be evaluated in this article, or claim that may be made by its manufacturer, is not guaranteed or endorsed by the publisher.
